# A cross-sectional survey of complementary and alternative medicine use by children and adolescents attending the University Hospital of Wales

**DOI:** 10.1186/1472-6882-6-16

**Published:** 2006-05-02

**Authors:** Nigel W Crawford, Domenic R Cincotta, Alissa Lim, Colin VE Powell

**Affiliations:** 1 Department of General Paediatrics, University Hospital of Wales, Heath Park, Cardiff CF14 4XW, UK; 2 Department of General Paediatrics, The Royal Children's Hospital, Melbourne, VIC 3052, Australia

## Abstract

**Background:**

A high prevalence of CAM use has been documented worldwide in children and adolescents with chronic illnesses. Only a small number of studies, however, have been conducted in the United Kingdom. The primary aim of this study was to examine the use of CAM by children and adolescents with a wide spectrum of acute and chronic medical problems in a tertiary children's hospital in Wales.

**Methods:**

Structured personal interviews of 100 inpatients and 400 outpatients were conducted over a 2-month period in 2004. The yearly and monthly prevalence of CAM use were assessed and divided into medicinal and non-medicinal therapies. This use was correlated with socio-demographic factors.

**Results:**

There were 580 patients approached to attain 500 completed questionnaires. The use of at least one type of CAM in the past year was 41% (95% CI 37–46%) and past month 26% (95% CI 23–30%). The yearly prevalence of medicinal CAM was 38% and non-medicinal 12%. The users were more likely to have parents that were tertiary educated (mother: OR = 2.3, 95%CI 1.6–3.3) and a higher family income (Pearson chi-square for trend = 14.3, p < 0.001). The most common medicinal types of CAM were non-prescribed vitamins and minerals (23%) and herbal therapies (10%). Aromatherapy (5%) and reflexology (3%) were the most prevalent non-medicinal CAMs.

None of the inpatient medical records documented CAM use in the past month. Fifty-two percent of medicinal and 38% of non-medicinal CAM users felt their doctor did not need to know about CAM use. Sixty-six percent of CAM users did not disclose the fact to their doctor. Three percent of all participants were using herbs and prescription medicines concurrently.

**Conclusion:**

There is a high prevalence of CAM use in our study population. Paediatricians need to ensure that they ask parents and older children about their CAM usage and advise caution with regard to potential interactions.

CAM is a rapidly expanding industry that requires further evidence-based research to provide more information on the effectiveness and safety of many CAM therapies. Statutory or self-regulation of the different segments of the industry is important. Integration of CAM with allopathic western medicine through education and better communication is slowly progressing.

## Background

### Definition

The use of complementary and alternative medicine (CAM) by children and adolescents is something that all health professionals involved in their care need to be aware of. CAM can be defined as, "diagnosis, treatment and/or prevention which complements mainstream medicine by contributing to a common whole, by satisfying a demand not met by orthodoxy, or by diversifying the conceptual frameworks of medicine" [1].

### Prevalence studies

A high prevalence of CAM use is well documented in children with a chronic illness, for example: oncology patients 42%, juvenile rheumatoid arthritis 70% and inflammatory bowel disease 72% [2-4]. Recent studies recorded significant usage of CAM in community paediatrics; 32% of children newly diagnosed with autism; 54% with behavioural problems and 56% in families of children with cerebral palsy [5-7].

Reported prevalence of use in adults ranges between 9–65% [8]. Fewer studies have been conducted in children, but a systematic review in 1999 found 9–70% of them used CAM. [9] More recent CAM studies in paediatric primary care showed a reported usage of 21–33 % [10,11] and hospital based studies 12–53%[12-14]. This study used the same methodology as a research team based at the Royal Children's Hospital in Melbourne, Australia [15]. Their study in 2002 found an overall yearly prevalence of CAM use of 51%.

Few surveys have been conducted in the UK, a study of adult cancer patients in 2001 found a yearly prevalence of CAM use of 49% [16]. A paediatric study by Simpson *et al*. from 1996 study in the Bath area found a general paediatric outpatient prevalence of 21% [17] and in a recent Leeds study, use ranged between 12–62%, in a sample which included healthy children and those with a chronic illness [18]. In a population based childhood study CAM use was 18% [19].

### Expenditure

It is an expanding industry; in 2000 MacLennan *et al*. repeated a 1993 representative population survey in South Australia [20,21]. Extrapolation of expenditure in 2000 was AUS$2.3 billion in Australia and US$34 billion in the U.S. population. In Australia this represented a 62% increase and the expenditure on alternative therapies was nearly four times the public contribution to all pharmaceuticals. There is little data on CAM expenditure in the United Kingdom, in 1998 90% of CAM provision was purchased privately with an annual out of pocket expenditure of approximately £450 million [22]. In 2003 it was determined that £211 million was spent on herbal therapies alone [23]. These figures are from adult based studies, making it difficult to identify expenditure on children, but it could be hypothesised that as family spending on CAM increases a proportion of this will be for use by children and adolescents.

### Public health

The use of CAM in the community at large, including children, is not just a clinical or hospital based issue. There are far reaching public health implications as highlighted by the House of Lords Report into CAM [24]. The American Journal of Public Health devoted an entire issue to complementary and alternative medicine in 2002 [25]. Especially important in children is the safety of CAM therapies, their potential for interactions and regulation of the CAM industry. The efficacy of these therapies is also a major issue, which further evidence-based research needs to address.

### Methodological limitations

A major limitation of the few studies conducted into CAM in children is the variable methodology and this study aims to address some of these limitations. Ernst only included only 10 of a possible 100 studies in a systematic review because of these issues [9]. Some fundamental problems with study design included the fact that most used a self-administered questionnaire, which has significant limitations, including problems of confusion of the overall meaning of questions and misinterpretation of individual terms or concepts that cannot be clarified. This is particularly important in the CAM field where definition and clarification is so important. Self-administered questionnaires may often be incomplete and have a poorer response rate than an interview administered questionnaire. The size of previous studies has also been variable, as has the country survey's have been conducted in. This combined with a variable definition of CAM makes direct comparisons difficult. The time frame of CAM use is also often not clear, with many using 'lifetime prevalence', that is CAM use at any point in the past. This is prone to recall bias. A discussion on adverse effects was not mentioned in 70% of the papers reviewed and 90% of did not clarify the cost of these therapies. The review was conducted in 1998, but many of the problems outlined above still exist, with surveys on CAM use in children often being self-administered questionnaires and include lifetime prevalences.

### Aims

The primary aim was to examine the use of CAM by children and adolescents in a tertiary children's hospital in Wales. This included those with acute and chronic illnesses. Secondary aims include: 1. Investigate association of CAM usage with socio-demographic factors, 2. Ascertain the frequency of CAM use, its cost and perceived helpfulness, 3. Explore patients'/parents' knowledge regarding the risks of side effects from CAM and their possible interactions with other therapies, 4. Identify whether CAM use is discussed with medical practitioners and whether inpatient medical records showed any evidence of appropriate documentation and 5. Examine the public health implications of CAM use

## Methods

A cross-sectional survey of the use of CAM by children and adolescents was undertaken from January to February 2004 at the University Hospital of Wales (UHW), Cardiff. This is a tertiary paediatric centre with approximately 10,000 inpatients and 6,500 outpatients seen per year (Allaway J, personal communication, 2005). The questionnaire was based on that used in an identical study at the Royal Children's Hospital, Melbourne, Australia [15].

The sample population of 500 participants were divided into 100 inpatients and 400 outpatients. The age range was between 0–18 years. The outpatients were also in groups of 100, namely: endocrinology, general paediatrics, gastroenterology and respiratory. These groups were chosen to produce a broad cross-section of the tertiary hospital population. The sample was obtained by interviewing consecutive inpatient admissions and children attending the relevant outpatient clinics over the study period. None of the paediatricians caring for children at UHW were practitioners of any form of CAM.

Data were collected in a structured interview-administered questionnaire with the patient and/or parents/guardian. It took approximately 10–15 minutes to complete. There were four interviewers involved in the study (NC, DC, AR & DW). Three of interviewers were paediatricians in training and the fourth (DW) a pre-registration pharmacist. All were calibrated prior to commencement of the study, by discussing as a group questionnaire administration and recording of data. The questionnaire had been piloted in Melbourne and some of the difficulties in administering the questionnaire were discussed with their research team. The questions were predominantly of a closed nature with multiple categories, including areas for comments. The interviewers were not involved in the medical treatment of the patients and did not enter into discussions regarding their personal opinions about CAM. Any specific questions regarding therapies were directed to their treating medical practitioner.

Exclusion criteria included insufficient knowledge of English and those children/adolescents admitted to intensive care. Written consent was obtained prior to commencing the interview. All patients/parents were given an information sheet. The South East Wales Local Research and Ethics Committee and the Research and Development committee at UHW approved the study.

### Definition

The type of CAM was divided into seven medicinal (naturopathic, herbal, homeopathic, Traditional Chinese medicine, health products, dietary and food supplements or non-prescribed vitamins and minerals) and thirteen non-medicinal (chiropractic, naturopathy, Traditional Chinese therapies, acupuncture, aromatherapy, iridology, therapeutic massage, reflexology, Buteyko breathing, kinesiology, reiki, hypnosis, special exercises or modified diet) therapies. There was also a free text area for CAM therapies not included on the list. Use of these therapies in the last year and past month was clarified. Non-prescribed vitamins and minerals were included as a CAM because a medical practitioner is not monitoring their use, despite them being taken as a medicine with therapeutic intent.

### Statistical considerations

In determining the sample size a literature review was performed to determine the expected frequency of CAM usage. This was estimated to range between 10 –70%. [9] A sample size of 100 for each group (500 subjects in total) will estimate any proportion to within +/- 10%, or better, with 95% confidence. This sample size is also identical to that used in the in Melbourne study [15]. The data was entered into a Microsoft Excel^© ^database and analysed using SPSS version 11.0^©^. Initial univariate analyses compared users and non-users of CAM. Proportions were compared using a Pearson chi-square test, with point estimate and 95% confidence intervals determined and a 'p value' of <0.05 considered statistically significant.

The data entry was checked by manually reviewing a computer generated random 5 percent of the data set at completion of the study. The stability of the questionnaire was reviewed by re-conducting the interview in a random 5 percent of respondents. This was done by telephone, 4–6 weeks after the initial interview. The interviewers were also compared, looking at any differences in CAM disclosure. Data has been collected and presented in an anonymous manner. All master copies of subject lists and questionnaires were retained securely within the Department of Paediatrics, UHW.

## Results

Five hundred and eighty patients were approached to complete 500 questionnaires. Five were excluded because of insufficient English language skills and 39 were missed because they left before being seen in busy clinics. Ten had already been seen in previous clinics or as inpatients and were therefore not re-interviewed. There were 23 who refused or did not fully complete the survey and 3 who did not have a legal guardian present. The interview was conducted with the index case (adolescent) in 7% of cases and their mother in 75%, father 17% and other guardian in 1%.

The study population of 500 interviewed was 52% male, with a mean age of 8 years (standard deviation 5.1 years) and a median of 7.8 years (range: 1 month to 19 years). The family income was greater than £20,000 for 52% of those studied. Tertiary education had been gained by 47% of mothers and 48% of fathers. Sixteen percent of families had private health cover that included cover of the index case.

Table 1. outlines 1-yearly CAM usage. The yearly prevalence of using at least one CAM was 206 out of 500 or 41%. The point prevalence of all CAM use in the last month was 26%. The prevalence of CAM use were very similar in the inpatients and the four different outpatient groups, except for being possibly slightly higher in the respiratory group (49%).

**Table 1:** 1-year Prevalence of Complementary and Alternative Medicine (CAM) Use

**Group**	**Prevalence (%)**	**95% Confidence Interval (%)**
Any CAM Use (overall)	41	37–46
Medicinal CAM	38	34–42
Non-medicinal CAM	12	9–14
Both medicinal and non-medicinal CAM	8	6–10
Excluding vitamins and minerals	25	21–29
CAM >/= 3 times (of total participants)	36	32–41
**Patient Group**		
Inpatients CAM use	39	30–49
Endocrine CAM Use	39	30–49
General Paediatric CAM Use	37	28–47
Gastroenterology CAM Use	42	33–52
Respiratory CAM Use	49	39–59

Table 2 outlines the socio-demographic factors associated with CAM use. Those using CAM had a higher family income and parental tertiary education. There were no differences in CAM use with regards to gender, age, number of children in the household or private health insurance.

**Figure 1:**
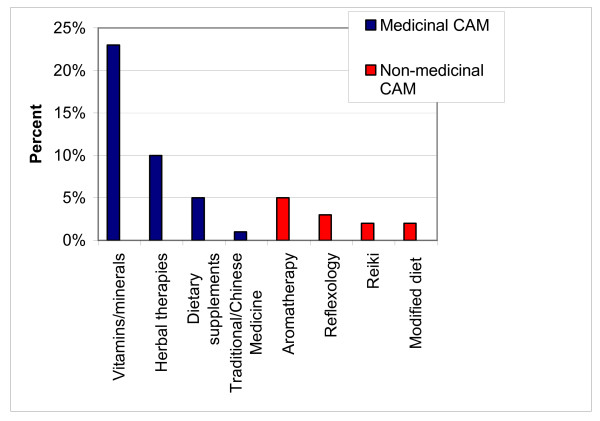
Most common types of CAM used- medicinal and non-medicinal (percentage of total participants)

The most commonly used types of CAM are outlined in Figure 1. The most frequently used medicinal CAM were non-prescribed vitamins and minerals and herbal therapies. In non-medicinal CAM group aromatherapy and reflexology were the most commonly used.

**Table 2:** Socio-demographic factors associated with CAM use

**Variable**	**CAM users/Total**	**Percent %**	**Pearson chi-square for trend (df = 1)**	**p value**
Demographics				
Age (yr)				
<2	33/74	45		
2 – 4	36/97	37		
3 – 8	48/103	47		
9 – 11	28/89	31		
> 12	61/137	46	0.27	0.60
				
Household Income (£)				
< 6000	8/37	22		
6001 – 12000	16/53	30		
12001 – 20000	45/109	41		
20001 – 30000	35/94	37		
> 30000	83/159	52	**14.30**	**<0.001**
				
No. of children in household				
One	49/120	41		
Two	89/205	43		
Three	41/101	41		
> Three	25/69	36	0.44	0.51
				
**Variable**	**CAM users/Total**	**Percent%**	**Odds Ratio (95% C.I.)**	**p value**
Sex				
Male	113/259	44	c	
Female	93/241	39	0.8 (0.6 – 1.1)	0.25
				
Country of birth (child)				
UK	201/485	41	c	
Overseas	4/13	31	0.6 (0.2 – 2.1)	0.44
Country of birth (mother)				
UK	186/455	41	c	
Overseas	18/42	43	1.1 (0.6 – 2.1)	0.80
Country of birth (father)				
UK	179/440	41	c	
Overseas	24/54	44	1.2 (0.7 – 2.1)	0.60
				
Post secondary education (mother)				
No	84/260	32	c	
**Yes**	**120/231**	**52**	**2.3 (1.6 – 3.3)**	**<0.001**
Post secondary education (father)				
No	92/249	37	c	
**Yes**	**109/234**	**47**	**1.5 (1.0 – 2.1)**	**0.03**
				
Private Insurance				
No	170/418	41	c	
Yes	34/77	44	1.2 (0.7 – 1.9)	0.57

c = comparative group

The use of prescription medications in the last month was 73% (358/493). The types of medications used were classified into the listed groups in Table 3. A total of 22% (108/493) used medications from more than one group. Five percent of participants had used herbal medicines in the last month and of those 68% (17/25) were also using a prescription medication. Thus 3% of participants overall were using herbs and prescription medications concurrently. Only 13% (32/256) of respondents knew there was the potential for interactions and of those, 56% (18/32) could actually specify one. Of those using CAM 5% (9/182) had experienced side effects. All of the side effects described were of a minor nature. The knowledge of any potential side effects of CAM was 15% (73/500) and 82 % (60/73) of those could name one. None of the 23 inpatients using CAM in the last month had this documented in medical or nursing notes.

**Table 3:** Use of prescription drugs in the past month. *Based on table by Madsen *et al*.[14] [N = 493]

	n	%
No drug use	135	27
Drugs against cardiac disease	8	2
Drugs against CNS disease (e.g. anticonvulsants)	22	5
Anti-neoplastic drugs (prednisolone, immunosuppressive drugs, folic acid)	36	7
Drugs against GI disease	71	14
Insulin or other hormones	71	14
Antibiotics and other systemic drugs against infectious diseases	99	20
Drugs for asthma, eczema, allergy	103	21
Other: Over the counter (OTC), paracetamol nutritional supplements/vitamins on doctors prescription	77	16

**Figure 2:**
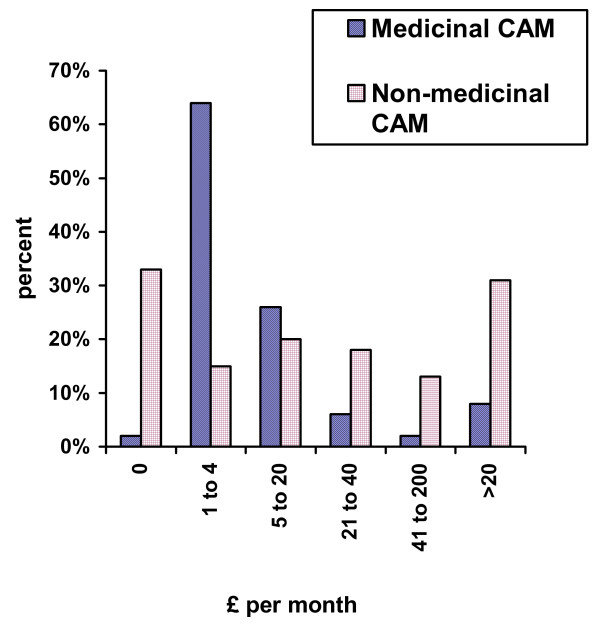
Expenditure on CAM (£ per month)

Expenditure is depicted in Figure 2. There is a trend towards higher expenditure in non-medicinal CAM, which had a higher median cost (£5–20 per month) than medicinal CAM (£1–4 per month).

CAM was self- initiated (parent/participant/family/friend) in 74%, with much lower initiation/recommendation by doctors (6%) or CAM practitioners (14%), At least one CAM was perceived as helpful by 57% (117/206) of CAM users. The use of CAM was not disclosed to the medical practitioner in 66% (135/206)of cases. 52%(97/185) medicinal and 38% medicinal CAM as safe (19%), natural (9%) and available over the counter (7%). Quotes included: "Doctors don't have the time" or are "Not interested", "I wanted to tell the doctor but feel that they don't want to hear", "doctors not bothered by colic", or the "doctor is ignorant so would not be interested".

The four interviewers were compared with regards to disclosure of CAM use in the past year as shown in Table 4. The random data check revealed a maximum error rate of 0.7% (95%CI 0.4–1.3%). The stability testing of the questionnaire showed a 3.3% (95%CI 2.5–4.4%) difference in responses when the survey was re-conducted over the telephone.

**Table 4:** CAM use disclosure by interviewer

Interviewer	Occupation	Number of CAM users/Total number interviewed	Percentage (95% confidence interval)
NC	Paediatric Specialist Registrar	73 out of 210	35% (29 – 41%)
DC	Paediatric Specialist Registrar	50 out of 127	39% (31 – 48%)
AR	Paediatric Senior House Officer	46 out of 100	46% (37 – 56%)
DW	Pharmacy student	37 out of 63	63% (46 – 70%)

## Discussion

### Prevalence

Our results with a yearly prevalence of 41% (95% confidence interval (CI) 37–46%) is in the upper range compared with studies worldwide [9]. Our general paediatric subgroup used 37% (95%CI 28–47%), which is higher than the general paediatric outpatient UK study in 1996 [17]. This may be a true increase in CAM use over that time, but confounders include; differences in the two outpatient populations, the definition of CAM used or the fact that our study was interview led, rather than a self-administered questionnaire, which may underestimate use. There was no statistical difference in the prevalence amongst the inpatient and outpatient groups with overlap of the 95% confidence intervals (Table 1). All of the four outpatients included patients with chronic illnesses and the high number of cystic fibrosis patients in the respiratory outpatients may partially explain their slightly higher prevalence. The prevalence in Cardiff is significantly lower the prevalence found in the Melbourne study (51%, 95% CI 47–56%) [15]. The reasons for this difference have been explored in a separate comparative analysis between the two tertiary centres [26].

### Socio-demographic factors

Socio-demographic factors associated with CAM use were parental tertiary education and high income. This supports the view that CAM is associated with higher socio-economic status [12,14]. Higher levels of education may lead to exposure to these therapies, especially given their high profile in the media. The lack of other socio-demographic factors implicated in CAM use from our study supports many of the previous surveys [11,13,27].

### Cost

Nearly two-thirds of medicinal CAM cost less than £5 per month, with about one-third spent nothing per month. This supports the assertion that CAM may not be as expensive as is commonly believed, as concluded by a large Canadian study which found that CAM was less than CAN$20 in 37% of cases and more than CAN$60 in only 6% [27]. A BBC commissioned survey in 1999 of CAM use amongst adults in the United Kingdom approximated CAM expenditure of £14 per month with about one-third spending less than £5 per month [28]. Non-medicinal CAM tended to be more costly as seen in this study, with about one-third spending more than £20 per month and 13% spending more than £40. This may limit their availability and therefore prevalence, which was significantly less than medicinal forms of CAM (12%. versus 41%). Of note some non-medicinal therapies were 'free' because treatments such as therapeutic massage or reiki were provided at no cost by friends or relatives.

### Disclosure

Disclosure of CAM use to medical practitioners is important if the issue is to be discussed and any potential interactions or adverse effects detected. In our study 66% did not disclose CAM usage to their doctor, which is similar to previous studies [15,18,29]. Reasons for lack of disclosure may reflect a perceived lack of acceptance by the physician. It is important part of doctor-patient communication that issues such as CAM is discussed in a nonjudgmental and open manner. The "don't ask, don't tell" status quo, [29] is also reflected in the fact that there was no documentation of recent CAM use in the inpatient notes. Poor communication is supported by other studies, which found few doctors (16%) ask about CAM use [30].

In a study of paediatricians and nurses attitudes to CAM, many using CAM themselves, but only 40% actually asked about CAM [31]. The main reason given for not discussing these therapies was a lack of confidence to do so because of minimal education about these therapies and the limited safety and efficacy data available.

### Efficacy and perceptions

A review of 19 surveys between 1982 to 1995, looking into the referral patterns of physicians to CAM practitioners, highlighted the fact that decisions need to be based on efficacy studies, rather than "regional economics and cultural norms", and "somewhere between over enthusiastic belief and stubborn disbelief is a balanced perspective that will help patients and advance medical science" [32]. The perception of parents and adolescents of doctors as not being interested was commonly found in this study, with quotes including: "they were not interested", "did not have the time" or "wanted to tell the doctor but feel that they don't want to hear".

### Integrative medicine

Integrative medicine refers to "looking at a broad range of therapies and considering those that have the best evidence of safety and effectiveness in the context of holistic care". [33] It has been strongly supported by The Prince of Wales's Foundation for Integrated Health [34] and is becoming an important component of medical education. Cohen provides a practical example of integrative care, focusing on the chronically ill, "as many of these patients could benefit from the services of CAM practitioners... and as evidence emerges that some CAM are effective then it becomes ethically impossible for the medical profession to ignore them". [35] Studies have found that CAM practitioners are keen on integration. [36]

### Safety

One of the major concerns for medical practitioners regarding CAM use is safety. Despite popular belief, CAM is not always "natural" and "safe" or free of side effects, and there are many case reports of adverse events in children. These include: death, neurological disability, organ failure and organ puncture[37]. Possible interactions between medicinal CAM, particularly herbal remedies and prescription medications, is well documented [38]. When questioned the majority (87%) of adolescents or parents in this study could not name a potential interaction. It was determined that there was a 3% risk of a participant in the study concurrently taking a herb and prescription medication. This is quite a small number in our hospital-based population and may mean that the interaction potential is less than previously believed. All ingested therapies, however, have the potential to interact so communication and reporting of interactions remains very important. Up-to-date information about CAM is becoming more available with pharmacists, local drug advisory services and some Internet resources useful [39,40].

### Evidence-based medicine

Funding is a major issue for producing quality evidence-based research into CAM as resources are still allocated predominantly through traditional biomedical channels and in 1996 only 0.08% of funding in medical sciences was going into CAM [41]. As stated by Ernst; "The scientific establishment criticises the paucity of data and weakness of CAM hypotheses with one breath and with their next breath they withhold the money that would be essential for changing this situation" [42].

### Regulation

Following the House of Lords Science and Technology Committee report on CAM in 2000, there was a push for some CAM practitioners such as Osteopaths and Chiropractors to follow the medical model of statutory regulation as seen with the General Medical Council [24]. Alternatives to statutory regulation are self regulation, with development of national standards regarding training and competence. Welsh *et al*. looked in depth at the tensions and difficulties in CAM practitioners seeking self-regulation [43]. The Prince of Wales's Foundation for Integrated Health has also played an important role in this area [34].

All professions are prone to rogue practitioners, such as those outlined in the study of Traditional Chinese therapists treating atopic eczema with topical remedies that were shown on analysis to contain high levels of potentially hazardous steroids [44]. In these situations it is important that the public sees effective and visible disciplinary procedures.

### Limitations of this study

There were a number of potential areas of bias in this study, namely: 1. Questionnaire 2. Interviewer, 3. Interviewee, 4. Sampling, 5. Language bias. The questionnaire itself may have produce bias towards the listed agents as it is not possible to include all CAM therapies and there was a free text area. This was minimised by extensive review of the literature and including 7 medicinal and 13 non-medicinal therapies in the interview led questionnaire. Of note 5 of the 20 therapies accounted for the majority of CAM use, with some CAM only being used by less than 2% of the study population.

Despite calibrating the interviewers with a view to obtaining consistent responses, it remains possible that inconsistencies in asking questions and recording answers occurred The interviewers [NC, DC, AH, DR] had variable amounts of CAM disclosed (Table 4) and in particular CAM use was much higher (63%) in the surveys conducted by DW. He was a pharmacy student whilst the other three interviewers were paediatric trainees. All interviewers were similarly attired, with no identifying features. DW interviewed only a small proportion of participants (13%).and none of the inpatient subgroup. DW may have had higher CAM disclosure because as the only pharmacist amongst the interviewers he may have been better at gaining information about medication use and CAM. Alternatively there could have been some potential bias in the parents/patients perceptions of the different interviewers, thus affecting disclosure. In particular, the role of the three doctors as interviewers may have led participants to feel that they would not approve of CAM use. This would produce an under-estimate of CAM use in our population. The fact that the interviews were conducted in a busy outpatient clinic and on the inpatient wards may have inhibited disclosure of CAM use in some cases.

Different responses may also be obtained when interviewing the patient or either parent about CAM. The knowledge and opinions regarding CAM may differ within the family. The mother (75%) was the person most commonly interviewed in the survey. The prevalence may be under-estimated in cases where a guardian with less knowledge about the child completed the questionnaire. This study asked about CAM use in the past year, which is prone to recall bias, as participants may find it difficult to remember exactly what therapies were used. Repeating the questionnaire over the phone showed that it had stability with only a 3.3% difference in responses. There was, however, no differentiation made between positive or negative differences and individuals may have started or stopped taking CAM in the two months between the two surveys. It is also possible that respondents were more educated about CAM after becoming involved in the study, affecting the responses in the repeat questionnaire.

A representative sample is difficult to obtain, but by approaching consecutive inpatient admissions and outpatient presentations, the potential for sampling bias was minimised. Only 80 cases were excluded and 10 of those were because they had been interviewed previously. Information was not obtained to directly compare the demographics or other details of the 80 excluded cases from the 500 respondents.

This study excluded a small number of subjects (5 in total) who had insufficient knowledge of English, leading to language bias. Whilst this is a small number overall, it may be expected from the literature and on general principles that this group is more likely to use CAM. Cost was a factor as this study was not funded and there were insufficient resources to provide questionnaires in different languages or provide independent interpreters. This study was also designed with the aim of doing a direct comparison of CAM use between Cardiff and Melbourne, so their methodology was replicated, including exclusion of those with insufficient English language skills. Whilst this strengthens the comparability of data it highlights the need for further research into specific ethnic groups and CAM subtypes utilised by them.

### Applicability of the study

As outlined previously methodologies used in CAM surveys vary, reducing the comparability of studies and hence the applicability of our study elsewhere. The study population was a tertiary paediatric group based at one location, comprising children with acute and chronic illnesses. The results whilst not applicable to the general population, may apply to other tertiary paediatric centres with similar demographics and case-mix. Well-conducted population based studies are require to ascertain the overall use of these therapies in children.

## Conclusion

This study confirms a high prevalence of use in our tertiary paediatric population. CAM cannot be ignored and medical practitioners need to be better educated about these therapies. As stated by Coulter, "CAM is here to stay and will continue to present challenges for conventional medicine of how to respond"[45]. Paediatricians need to specifically ask about CAM use and document this appropriately. It should be discussed with patients in an open, non-judgemental way. In particular patients and medical practitioners should be aware of potential interactions and liaise with their pharmacy or drug advisory department if required. This will help patients and their parents make more informed, safe choices. Further evidence-based studies are required to provide more information on the effectiveness and safety of many CAM therapies [46]. The public health implications of CAM are far reaching and include regulation of the industry and protection of the public through appropriate safety and monitoring measures. Integrative medicine is slowly progressing bringing together western allopathic medicine and CAM therapists through improved communication and education.

## Competing interests

The author(s) declare that they have no competing interests.

## Authors' contributions

NC was an interviewer and co-author of the drafted manuscript. DC was also an interviewer and co-author of the drafted manuscript. AL provided background information and the questionnaire on which this study was based [15]. CP conceived of the study and was supervisor of the project. All authors read and approved the final manuscript.

## Pre-publication history

The pre-publication history for this paper can be accessed here:


